# Laboratory Measurement and Analysis of the Deteriorated Layer Permeability Coefficient of Soil-Cement Deteriorated in a Saline Environment

**DOI:** 10.3390/ma12142245

**Published:** 2019-07-12

**Authors:** Qing Jin, Xinzhuang Cui, Junwei Su, Tu Lu, Jieru Wang, Ruonan Han

**Affiliations:** School of Civil Engineering, Shandong University, Jinan 250061, China

**Keywords:** soil-cement, corrosion, deteriorated layer, permeability coefficient

## Abstract

The deterioration of soil-cement in a saline environment leads to a reduction in strength and an increase in permeability. Effective methods of determining the deteriorated layer permeability coefficient of soil-cement are currently lacking. A laboratory test method for measuring the permeability coefficient of the deteriorated layer was proposed using the modified permeability coefficient testing apparatus. According to the proposed method, the permeability coefficient of the deteriorated layer could be obtained after testing the permeability coefficient of the soil-cement specimen in acuring room and testing the equivalent permeability coefficient and deterioration depth of the soil-cement specimen in a deteriorated environment. Using the marine dredger fill from Jiaozhou Bay as a case study, the deteriorated layer permeability coefficients of soil-cements with different cement contents were tested. It turned out that the permeability of the deteriorated layer increases with age. At the beginning of the curing age, higher cement content led to a smaller permeability coefficient of the deteriorated layer of soil-cement. As the curing age increased, the deteriorated layer permeability coefficient of the soil-cement with higher cement content increased. The evolution of the permeability coefficient of a deteriorated layer with age can be formulated as the Logistic function. This study provides support for anti-permeability designs of soil-cement structures in saline environments.

## 1. Introduction

A soil-cement mixture is proportionally mixed and evenly stirred with cement, soil, water, and admixtures. In general, soil-cement, which is widely applied in ground reinforcement and underground construction seepage control projects, is characterized by high strength and low permeability [1,2]. However, in some cases, such as landfills, coastal sedimentary soils, and marine dredger fills, the ground water and ground soil are highly corrosive. Soil-cement in a corrosive environment for a long time period will inevitably deteriorate. This deterioration significantly reduces strength and increases the permeability coefficient, which has adverse effects on strength properties and service life.

Terashi et al. first proposed the concept of soil-cement deterioration and found that the deterioration decreased the effective diameter of the soil-cement pile [3]. A miniature penetration test was employed to measure deterioration depth and analyzed in the laboratory [4]. Hayashi investigated properties of the treated soil columns constructed by the dry method of deep mixing (DJM)about 17 years ago. Continuous core samples were taken to compare the physical properties and unconfined compressive strengths with those obtained 17 years ago [5]. Based on the field tests, Lee et al. and Terashi et al. analyzed the distribution of calcium content in the soil-cement pile and found that it agreed well with the distribution of soil-cement strength [6,7]. Yang et al. employed a miniature penetration test to study the relationships between the strengths of soil-cements maintained in curing rooms and seawater. The results show that the deterioration extends more quickly in seawater environments. With the increase in cement content, the strength of the cement-stabilized soil increased obviously, but the deteriorated depth reduced significantly [8,9]. It was pointed out that the strength reduction of soil-cement is related to the calcium ion leaching phenomenon. Ikegami et al. delivered an observation of a large-scale reinforced soil-cement body with an age of 20 years [10]. The deterioration depth reached 30 mm, and the calcium content and strength of the soil-cement were noticeably reduced. Nishada et al. proposed the leaching prediction model of calcium [11]. Kitazume et al. carried out a series of laboratory strength tests of soil-cement with an age of 1 year under various curing conditions, and the results showed that the unconfined compressive strength of soil-cement increases approximately linearly with the logarithm of time [12]. Cui proposed a prediction method of long-term bearing capacity of the soil-cement in a corrosive environment [13]. In addition, Huang studied the crystalline swelling mechanism of sulfate attack in soil-cement [14].

Some researchers have studied the permeability of soil-cements. Chew et al. compared the permeability of soil-cement with undisturbed soil [15]. Tests performed by Brandl revealed that the permeability coefficients of soil-cement decrease with time [16]. However, this research did not include the permeability of deteriorated soil-cement. Hara et al. investigated the influence of seawater on the properties change of lime-treated soil by laboratory experiments. A dissolution test, cone penetration test, moisture ratio test, and elemental analysis were performed on lime-treated soil specimens immersed in artificial seawater, and the softening of treated soil and changes in physical and chemical properties were examined [17]. However, Hara’s work was mainly focused on the properties of a deteriorated specimen, which is highly related to specimen size. Therefore, a main technical challenge is how to use the equivalent permeability coefficients obtained in a laboratory to design different soil-cement structures, such as a diaphragm wall composed of soil-cement piles. In reality, the anti-permeability reduction of a soil-cement structure in a corrosive environment results from the increase in the deteriorated layer permeability coefficient, which is not related to structure size. After acquiring the coefficient of deteriorated layer permeability in soil-cement, the equivalent permeabilities of deteriorated soil-cement structures with different sizes can be obtained. However, laboratory test methods to find the deteriorated layer permeability coefficient of soil-cement are currently lacking.

Based on Darcy’s law, this study proposed a novel laboratory test method to find the deteriorated layer permeability coefficient of soil-cement and modified the existing test apparatus. Using the marine dredger fill from Jiaozhou Bay in China as an example, the deteriorated layer permeability of soil-cement was tested and analyzed, and a prediction model of long-term permeability was preliminarily proposed. This study provides technical support for anti-permeability designs of soil-cement structures in corrosive environments.

## 2. Calculation Method of the Deteriorated Layer Permeability Coefficient of Soil-Cement

**Figure 1:**
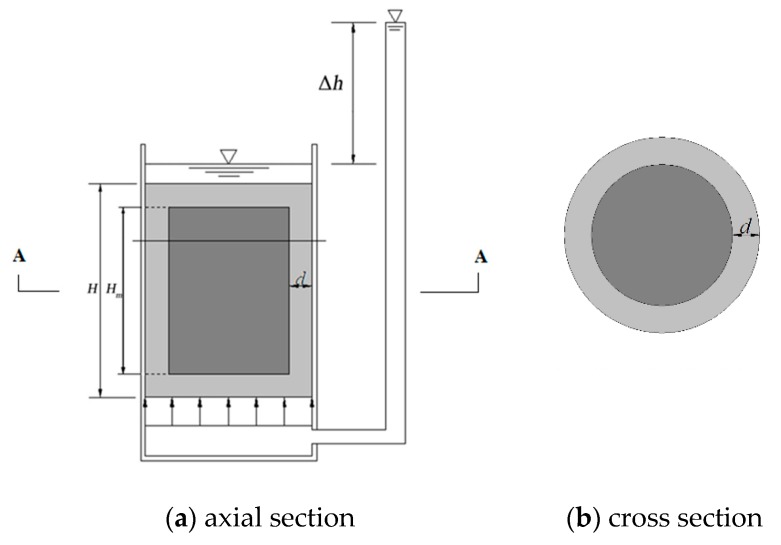
Soil-cement specimen.

Figure 1 shows the cylindrical deteriorated soil-cement specimen in a laboratory permeability test. The light gray area represents the deteriorated layer with depth d, and the dark gray area represents the non-deteriorated region. For the specimen in Figure 1, the permeability of the deteriorated layer can be derived according to Darcy’s law. Because the seepage flow is the same through any cross section of the specimen, the following equation can be obtained:

(1) $$v=k_{d}i_{t}=k_{d}i_{b}=k_{m}i_{m}=k_{c}i_{c}=k_{c}\frac{\Delta h}{H}=k_{c}\frac{\Delta h}{H_{m}+2d},$$

where *v* is the equivalent seepage velocity; *k_d_* is the permeability coefficient of deteriorated layer; *k_c_* is the equivalent permeability coefficient of the entire deteriorated specimen; *k_m_* is the equivalent permeability coefficient of the middle section of the specimen; *i_t_*, *i_b_*, *i_m_*, and *i_c_* are the hydraulic gradients of the upper deteriorated layer, the lower deteriorated layer, the middle section, and the entire deteriorated specimen, respectively; *H* is the total height of the specimen; *H_m_* is the height of the middle section; Δ*h* is the total hydraulic head loss; and *d* is the deterioration depth of the cement-soil.

The total hydraulic head loss equals the sum of the hydraulic head loss through each part of the specimen:

(2) $$i_{t}d+i_{b}d+i_{m}H_{m}=i_{c}H.$$

Because *i_t_* = *i_b_*, Equation (2) can be rewritten as follows:

(3) $$i_{t}=\frac{i_{c}H-i_{m}H_{m}}{2d}.$$

According to Equations (1) and (2), the permeability coefficient (*k_d_*) of the deteriorated layer can be calculated as follows:

(4) $$k_{d}=\frac{2k_{c}k_{m}d}{k_{m}H-k_{c}H_{m}}.$$

The middle section consists of the non-deteriorated and deteriorated regions (Figure 1). The seepage flow through cross section A–A (Figure 1) can be expressed as

(5) $$Q_{m}=k_{m}i_{m}A=k_{0}i_{m}\left(A-A_{d}\right)+k_{d}i_{m}A_{d},$$

where *Q_m_* is total seepage flow; *A* is the total cross-sectional area; *A_d_* is the cross-sectional area of the deteriorated layer; and *k_0_* is the permeability coefficient of the internal non-deteriorated region of the soil-cement.

Equation (5) can be rewritten as follows:

(6) $$k_{m}=k_{0}\left(1-R_{a}\right)+k_{d}R_{a},$$

where *R_a_* is the cross-sectional area deterioration rate of the soil-cement specimen.

By substituting Equation (6) into Equation (4), a quadratic equation in terms of *k_d_* can be obtained:

(7) $$R_{a}k_{d}^{2}+\left(k_{0}-k_{c}-k_{0}R_{h}R_{a}+2k_{c}R_{h}-2k_{c}R_{a}R_{h}\right)k_{d}-2k_{0}k_{c}R_{h}\left(1-R_{a}\right)=0,$$

where *R_h_* is the ratio of deterioration depth and to the total height of the specimen, *R_h_* = *d/H*.

Solving Equation (7), *k_d_* can be obtained as

(8) $$k_{d}=\frac{A+\sqrt{B}}{2R_{a}},$$

(9) $$(2k_{c}+k_{0})R_{\;a}R_{\;h}+k_{c}-2k_{c}R_{\;h}-k_{0},$$

(10) $$\begin{array}{l} B=k_{0}{}^{2}\left(R_{a}{}^{2}R_{h}{}^{2}-2R_{a}R_{h}+1\right)+2k_{0}k_{c}\left[R_{a}R_{h}\left(2R_{a}R_{h}-2R_{h}-4R_{a}+3\right)+2R_{h}-1\right]+\\ k_{c}{}^{2}\left[4R_{a}R_{h}\left(R_{a}R_{h}-2R_{h}+1\right)+4R_{h}\left(R_{h}-1\right)+1\right] \end{array}$$

## 3. Improvement of the Soil-Cement Permeability Testing Apparatus

**Figure 2:**
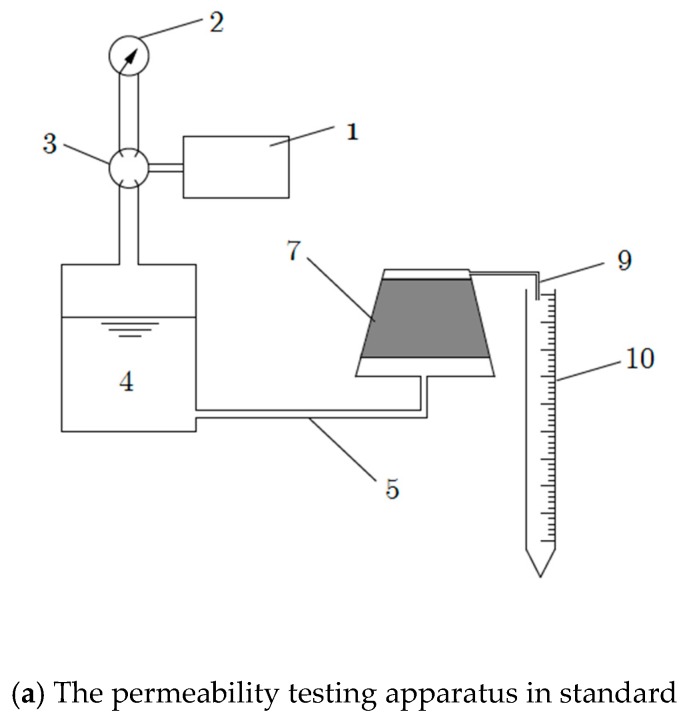

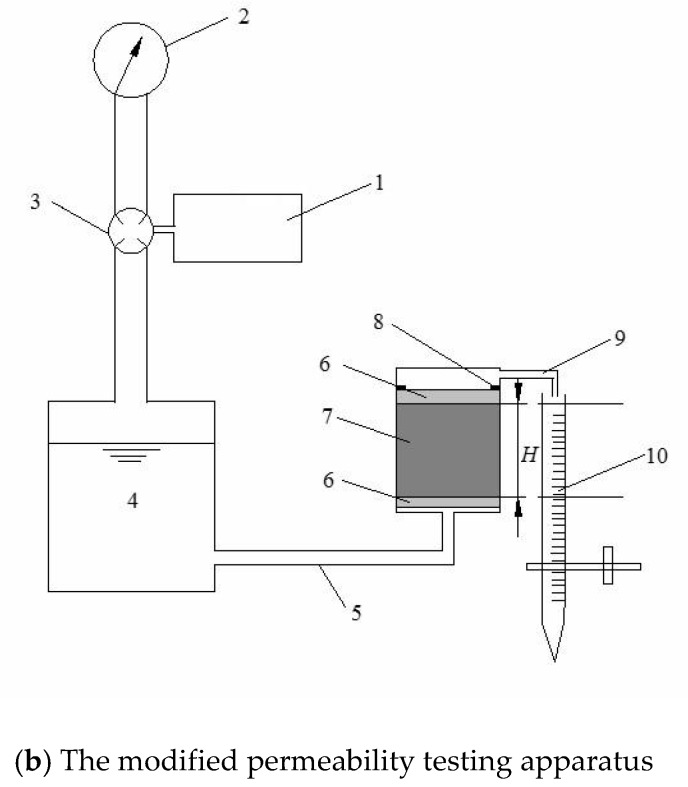
The modified permeability testing apparatus. 1: Air bump; 2: Pressure gage; 3: Pressure regulating valve; 4: Flume; 5: Water inlet; 6: Porous stone; 7: Soil-cement specimen; 8: Steel flake; 9: Water outlet; and 10: Burette.

**Figure 3:**
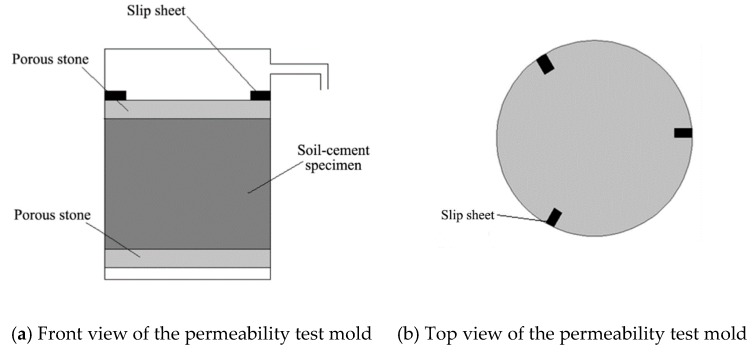
Permeability test mold.

According to the Specification for Mix Proportion Test of Cement-mixed Soil (JGJ/T 233-2011) [18], a truncated cone specimen is used for the permeability test. However, Equation (8) only applies to a cylindrical specimen and the permeability of the deteriorated layer cannot be measured using existing permeability testing apparatus. Therefore, in this study, the existing soil-cement permeability testing apparatus was modified as shown in Figure 2. The permeation mold was modified to a cylinder, and three steel flakes were welded onto the mold to prevent the cylindrical soil-cement specimen from sliding up (as shown in Figure 3). The modified testing apparatus of soil-cement permeability is suitable for a cylindrical specimen of 75 mm (diameter) × 30 mm (height), eliminating the need for a frustoconical specimen and simplifying the procedure. Before being piled into the mold, the specimen should be wiped with a wrung-wet cloth, and the side surface should be evenly smeared with grease. Porous stones are placed at the top and bottom of the mold.

## 4. Tests of Soil-Cement

Using the marine dredger fill from Jiaozhou Bay in China as a case study, the deteriorated layer permeabilities of soil-cements with different cements were tested.

### 4.1. Soil

The soil used in the tests was marine dredger fill taken from Jiaozhou Bay (pH = 8.20). The basic physical parameters and major ionic content are listed in Table 1 and Table 2, respectively. The soil is characterized by a high liquid limit, high moisture content, and hypersalinity. Table 3 shows major ions in the seawater.

### 4.2. Preparation and Maintenance of the Soil-Cement Specimen

Ordinary Portland cement grade 42.5 was used in the experiment. As shown in Figure 2, the natural moisture content of the marine dredger fill from Jiaozhou Bay was close to 1.5 times the liquid limit, so it was not necessary to add water when stirring. The soil-cement formulation consisted of three different cement contents (7%, 10%, and 15%). Some research suggests that the best method of stabilization of the soil could be a combination of mechanical compaction and chemical stabilization by cement. However, in this paper, specimens were placed directly in the curing room after the cement-soil slurry was poured in the mold, and compaction was not carried out to facilitate the forming process of specimens. For each cement content, two types of specimens were prepared:

(1) The first type of specimen was used for permeability tests. The diameter of the specimen was 75 mm and the height was 30 mm. Specimens were demolded after 24 h of curing. When the soil-cement specimen was cured in saline solution, the surface of the specimen contacted the saline solution directly. Therefore, the corrosion of soil-cement originated from the surface. The internal portion of soil-cement did not experience corrosion until the saline solution penetrated into that region. In this paper, specimens were cured under dry conditions in a curing room environment and under wet conditions in seawater. During immersion in seawater, the internal region of the soil-cement specimen did not experience corrosion from the saltwater and could be regarded as soil-cement in a curing room environment [19]. For the immersion maintenance case, the specimens were first maintained in a curing room for seven days and then in the seawater that was taken from the field. The characteristics of the seawater are shown in Table 3. The temperature of the seawater was kept at 20 ± 2 °C, and during the curing period, the concentration of ions in the seawater was measured every three days and the seawater was renewed to ensure the relative balance of the concentration of ions in seawater.
(2) The second type of specimen was used for tests of deterioration depth, which was needed for Equation (8). First, the soil-cement was filled into a cutting ring smeared with Vaseline. The cutting ring’s height was 20 mm and its diameter was 61.8 mm. Then, soil-cement specimens were maintained together with the cutting rings in a curing room and in seawater before they were demolded and tested, respectively.

### 4.3. Tests of Deterioration Depth

**Figure 4:**
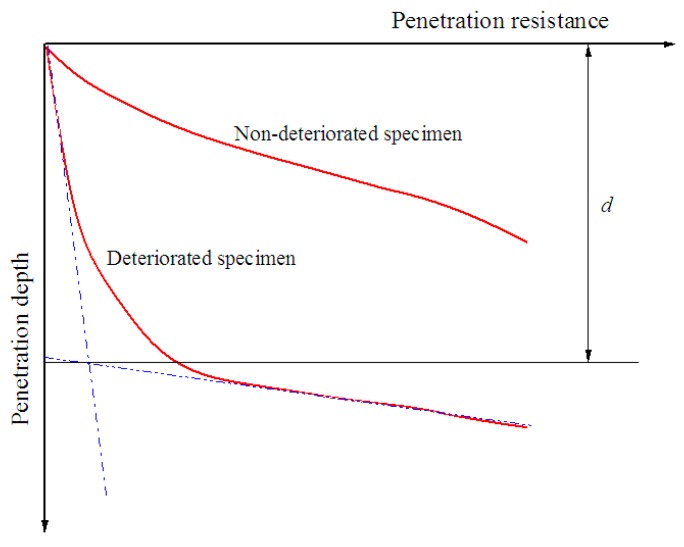
Penetration resistance-depth curves.

The miniature penetration test in the paper was carried out with reference to Yang's method. A miniature penetration test was employed to measure deterioration depth. The cone and cone shaft of the penetrometer were both made of stainless steel. The diameters of the cone and the cone shaft were 7 mm and 6 mm, respectively. The cone angle was 60°. The maximum penetration depth was 130 mm. Penetration resistance and penetration depth were displayed by a digital monitor. The test penetration speed was set as 1 mm/min. The penetration resistance-depth curves of deteriorated and non-deteriorated soil-cement were quite different, as shown in Figure 4. Compared with non-deteriorated soil-cement, the deteriorated soil-cement showed two obvious sections on the curve. The resistance remained low at the initial stage but had a sudden increase as the penetration depth increased to a certain value. Hara et al. defined the depth corresponding to the intersection of the extension lines of two sections as the deterioration depth, as shown in Figure 4. In this study, Hara’s definition method was adopted to obtain the deterioration depth of soil-cement [17].

### 4.4. Tests of Permeability

Using the modified permeability testing apparatus for soil-cement, permeability tests were carried out on specimens with different curing ages (28, 45, 60, 75, and 90 days) and curing conditions (curing room and seawater). The test procedure was as follows:

(1) Remove the soil-cement specimens from different curing conditions and wipe their surface with a wrung-wet cloth. Smear the specimens with grease and place them in the mold with one porous stone installed at the top and one at the bottom. Place the mold in the permeameter and cover the upper surface with filter paper.
(2) Increase the hydraulic pressure step-by-step. At the beginning of the experiment, the rate of hydraulic growth was 0.02 MPa per step until the hydraulic pressure reached 0.1 MPa, at which time the growth rate changed to 0.1 MPa per step. After applying each pressure level, the pressure should be stabilized for one hour before applying the next level of pressure until the water overflows.
(3) Record the hydraulic pressure and seepage volume after the fluid level in burette stabilizes. For specimens with large seepage volume, read the scale at an interval of 3–5 min; for specimens with small seepage volume, read the scale at an interval of 30–60 min. Take the average of the three groups of data for each specimen.
(4) Monitor the seepage process of the specimens during tests. If the water oozes from the lateral surface, the experiment should be stopped, and the specimens should be resealed before proceeding.

The permeability coefficient can be calculated from Equation (11):

(11) $$k=\frac{\gamma_{w}hV}{pA\Delta t},$$

where *k* is the permeability coefficient of the soil-cement specimen; Δ*t* is the time interval; *A* is the cross-sectional area of the specimen; *h* is the specimen height; *V* is the volume of the water through the specimen during the time interval (Δ*t*); *p* is the seepage pressure; and *γ_w_* is the unit weight of water.

From the permeability tests, the equivalent permeability coefficient of the deteriorated specimen cured in seawater (*k_c_*) and the permeability coefficient of the non-deteriorated specimens cured in a curing room (*k_0_*) can be obtained. Substituting *k_0_*, *k_c_*, and *d* into Equation (8), the permeability coefficient of the deteriorated layer (*k_d_*) can be calculated.

## 5. Test Results and Analyses

### 5.1. Deterioration Depth

**Figure 5:**
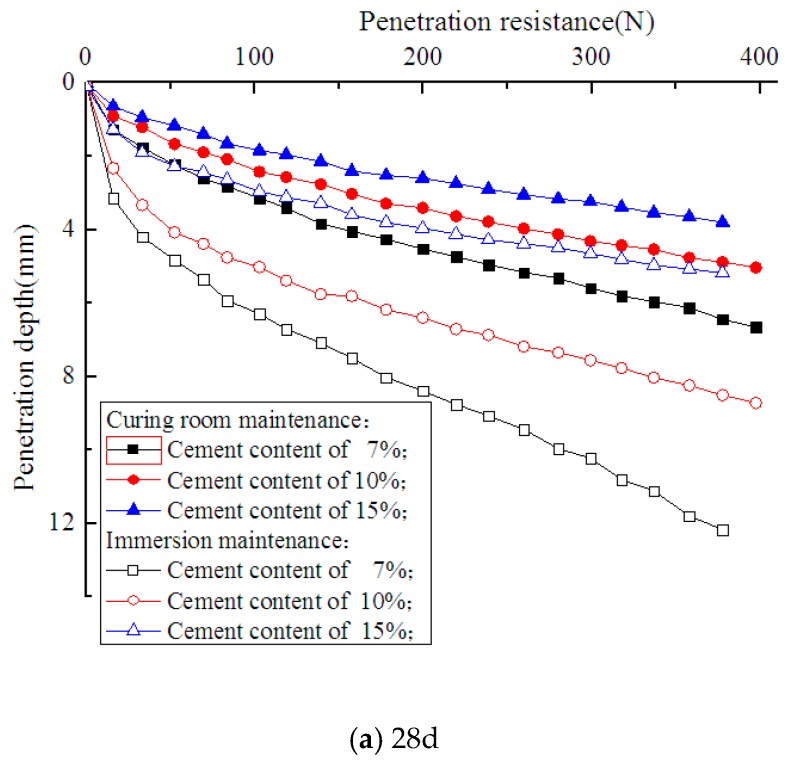

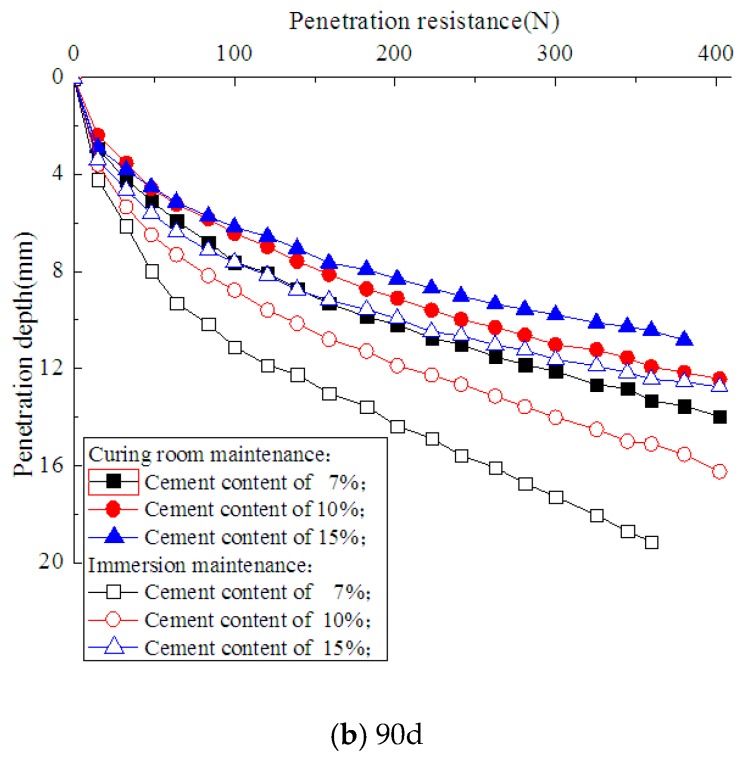
Penetration resistance-depth curves.

From the penetration tests on the soil-cement specimens in cutting rings with different curing ages and cement contents, the penetration resistance-depth curves were measured experimentally, as shown in Figure 5.

**Figure 6:**
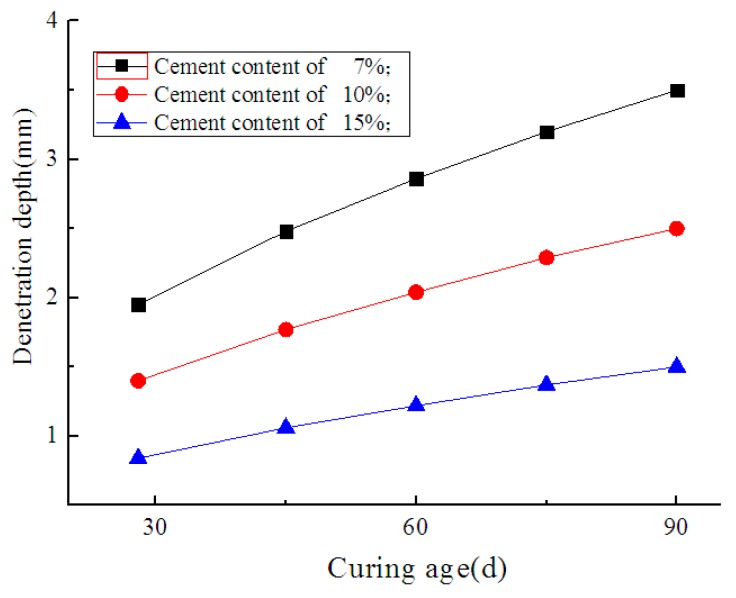
Variation curves of deterioration depth with curing age.

**Figure 7:**
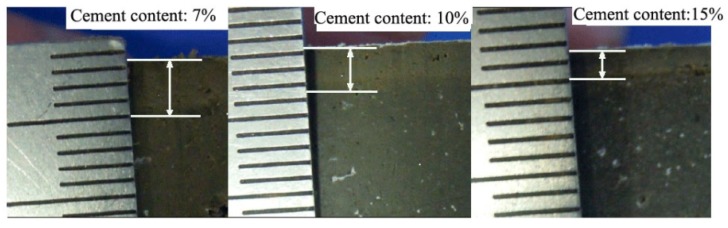
Axial section of the specimen maintained in seawater with a 90-day curing age.

Compared with the specimens from a standard curing condition, the specimens maintained in seawater demonstrated lower resistance at the same penetration depth, which indicates the occurrence of deterioration. Under the same curing conditions, a lower cement content corresponded to decreased penetration resistance. The deterioration depths of the specimens with cement contents of 7%, 10%, and 15%, and curing ages of 28, 45, 60, 75, and 90 days, were measured experimentally, as shown in Figure 6. Figure 7 shows the axial section of a specimen maintained in seawater with a 90-day curing age. As shown, the interior of the specimen remained dark, but its surface layer became lighter due to seawater corrosion. The deterioration depths of the specimens with cement contents of 7%, 10%, and 15% were 3.5 mm, 2.5 mm, and 1.5 mm, respectively, which is consistent with what is shown in Figure 6. It is indicated that as curing age increases, the growth rate of the deterioration depth of soil-cement specimens gradually decreases, while at the same curing age, deterioration depth increases with an increase in cement content. This proves the results of previous research showing that the durability of soil-cement can be improved by increasing cement content [19].

Ikegami et al. summarized the deterioration depths of soil-cement tested in the laboratory and measured in the field under varying environmental conditions (seawater, salty groundwater, and marine clay). The relationship between the deterioration depth and age appeared to be linear on a log–log graph, and the slope was approximately 0.5. Therefore, in this study, the relationship of the deterioration depth and age can be expressed as

(12) lgd=a+0.5lgt,

where *d* is the deterioration depth of soil-cement in mm; *t* is the curing age in days; and *a* is a constant.

**Figure 8:**
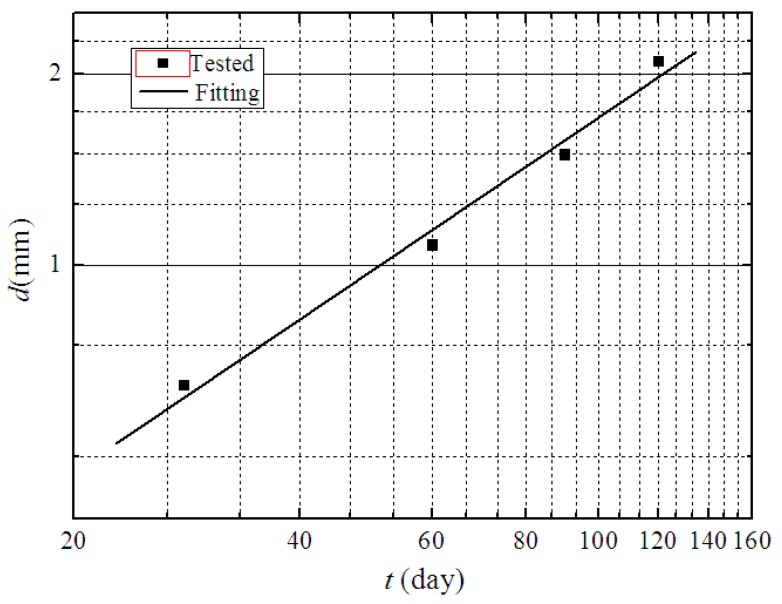
Fitting curve of deterioration depth of soil-cement with age.

The deterioration depth of soil-cement with a cement content of 15% was fitted with Equation (12), as shown in Figure 8, and the constant a= −0.82 was obtained. Equation (12) can be rewritten as follows:

(13) $$d=0.20\sqrt{t}(R=0.90),$$

where *R* is the correlation coefficient corresponding to the fitting precision.

**Figure 9:**
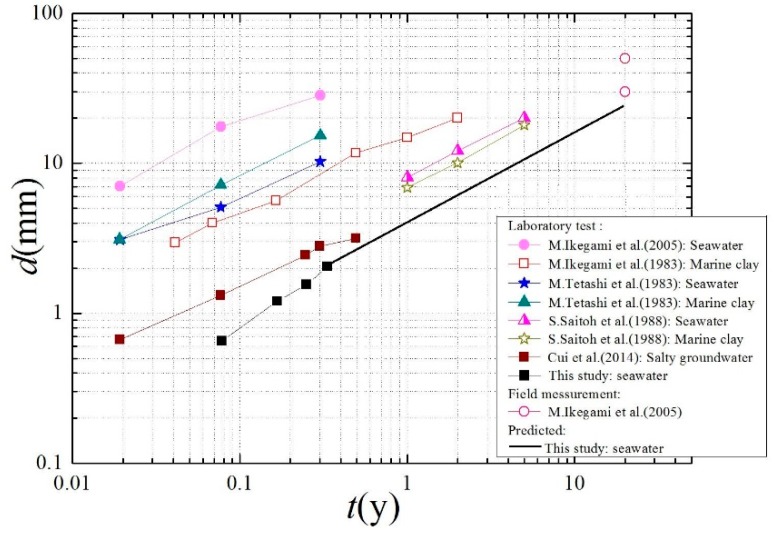
Variations of deterioration depths of different soil-cements with age.

The deterioration depths of soil-cement obtained by different researchers and measured and predicted in this study are shown in Figure 9 [3,4,13]. It can be seen that the deterioration depth in this study is lower than depths from other test results.

### 5.2. Permeability Coefficient of Deteriorated Layer

**Figure 10:**
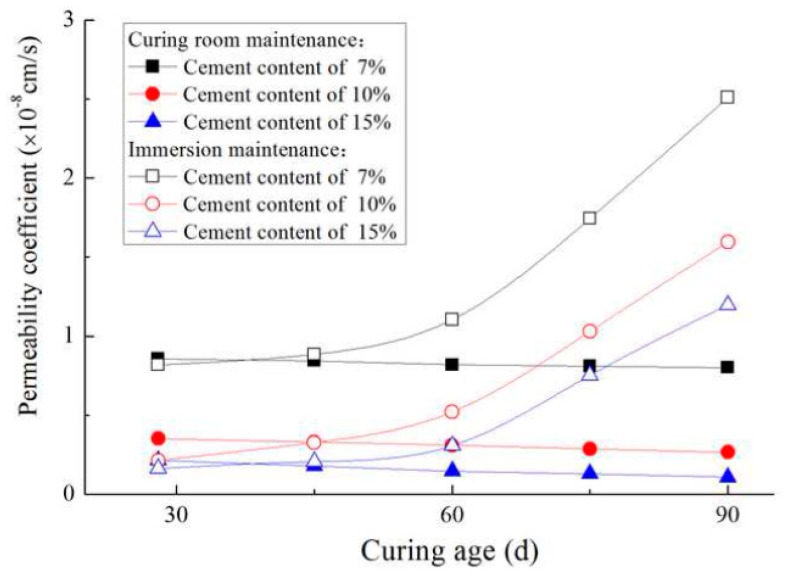
Variation curves of permeability coefficients with curing age.

**Figure 11:**
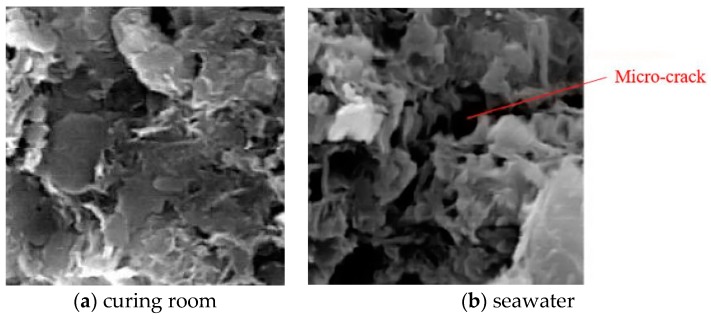
SEM pictures of soil-cement. (**a**) Curing room and (**b**) seawater.

Figure 10 shows the permeability coefficients of specimens under different curing conditions with different curing ages. As shown, the permeability coefficients for curing room maintenance decreased with an increase in curing age and cement content. Under the curing condition of seawater, the growth rate of permeability coefficients increased with curing age. When the curing age was shorter (such as 28 days), the permeability coefficient of soil-cement under immersion maintenance was smaller than that maintained in a curing room. However, with increasing age, the permeability coefficient of soil-cement under immersion maintenance was higher than that maintained in a curing room. This is mainly related to the corrosion mechanism of SO_4_^2−^ in seawater. The sulfate ions in the seawater react with the calcium hydroxide produced by the hydration of the cement to form gypsum. The gypsum reacts with the calcium sulfoaluminate and the aluminum colloid to form ettringite. The ettringite was expandable, which plays a positive role in reducing the permeability of the cement soil. And this can be proved by previous studies [20,21,22]. However, as the curing age increased, a large amount of ettringite was produced in the soil-cements, which makes them loose and causes micro-cracks, as shown in Figure 11b. Figure 11 shows SEM images of the soil-cement with a cement content of 15% and a 90-day curing age under various curing conditions. Microcracks on the surface of soil-cement resulted in an increase in permeability.

**Figure 12:**
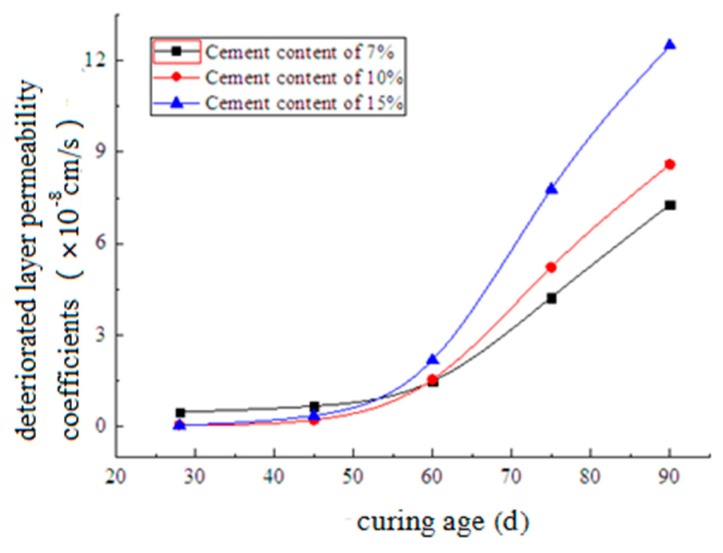
Development of the deteriorated layer permeability coefficients.

Figure 12 shows the relationship between the deteriorated layer permeability coefficients and curing age. As shown, with a short curing age, a higher cement content corresponded with a smaller permeability coefficient. However, when the curing age was over approximately 60 days, the situation was reversed. This is mainly because of the expansive ettringite generated by the reaction between the SO_4_^2−^ in seawater and the components of soil-cement. A higher cement content corresponded with greater ettringite production. The ettringite filled into the pores in soil-cement and reduced its permeability. However, excess ettringite expanded the deteriorated layer of soil-cement specimens with longer curing ages and resulted in their fracture. Therefore, the permeability of soil-cement with high cement content was larger than that with lower cement content.

**Figure 13:**
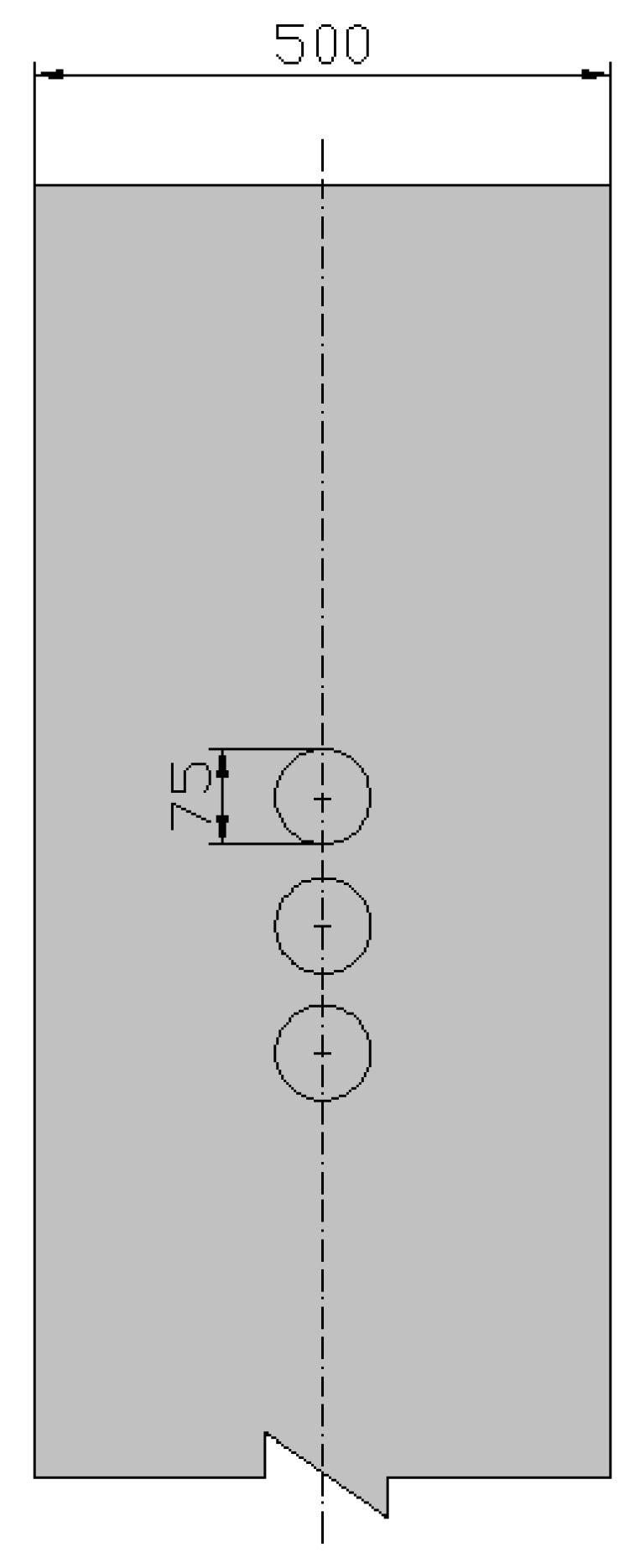
Sampling points (unit: mm).

In addition to early permeation tests in the laboratory, the long-term permeability coefficient of the deteriorated layer of a soil-cement pile constructed 20 years ago at Qingdao Port was measured. The cement content of the pile was 15%. Three core samples were collected from the lateral surface of the pile 1 m under the groundwater level, as shown in Figure 13. Long-term permeability coefficients were measured after smoothing both ends of the samples. The results for the three samples were 13.96 × 10^−8^ cm/s, 13.70 × 10^−8^ cm/s, and 14.52 × 10^−8^ cm/s, with an average value of 14.41 × 10^−8^ cm/s.

**Figure 14:**
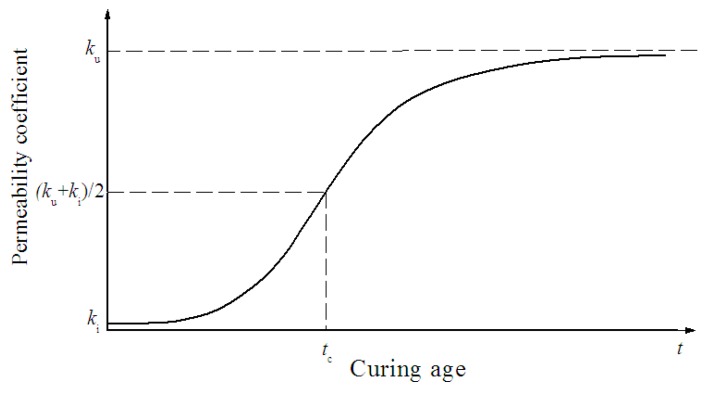
Theoretical evolution of the permeability coefficient of the deteriorated layer.

Figure 13 shows that with a 90-day curing age, the permeability coefficient of the deteriorated layer steadily increased. However, in theory, it is not possible for the permeability coefficient to increase without limit. It should approach a stable value, as shown in Figure 14. The curve in Figure 14 can be formulated as a logistic function:

(14) $$k_{d}=\frac{k_{i}-k_{u}}{1+{\left(\frac{t}{t_{c}}\right)}^{p}}+k_{u},$$

where *k_d_* is the permeability coefficient of the deteriorated layer; *k_i_* is the initial permeability coefficient; *k_u_* is the ultimate permeability coefficient; *p* is a constant reflecting the rate of deterioration; and *t_c_* is the curing age when the permeability coefficient equals (*k_u_*+*k_i_*)/2, as shown in Figure 14.

**Figure 15:**
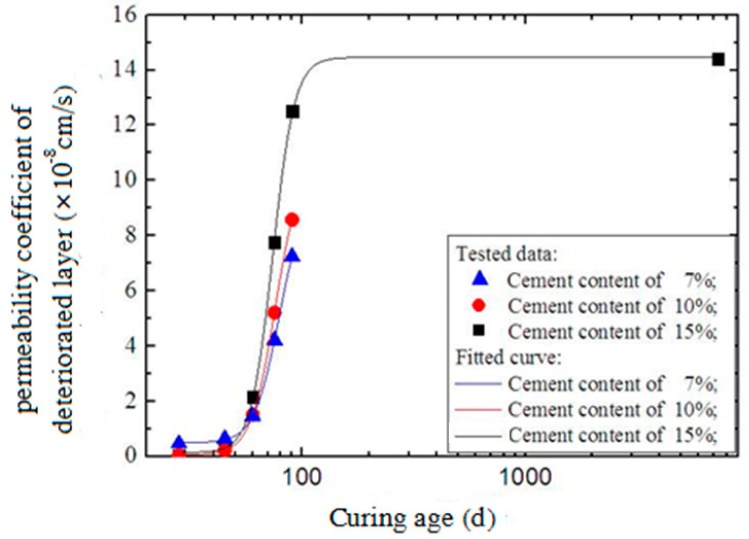
Fitting curves of the permeability coefficient of the deteriorated layer.

The tested permeability coefficients of deteriorated layers were fitted with Equation (14), as shown in Figure 15. The curve fit agreed well with the test data. The fitting parameters are listed in Table 4. It is shown that higher cement content leads to a larger ultimate permeability coefficient. In addition, higher cement content caused a larger value of p, indicating that higher cement content leads to a higher deterioration rate.

Equation (14) is the prediction model of the permeability coefficient of a soil-cement deteriorated layer. The model can be applied in anti-permeability designs of soil-cement structures in a saline environment.

## 6. Application of the Prediction Model for the Deteriorated Layer Permeability Coefficient

The diaphragm wall composed of soil-cement piles is widely used in coastal anti-seepage engineering. However, the soil-cement diaphragm wall experiences constant deterioration due to corrosion from sea water and salty groundwater, leading to an increase in the permeability coefficient. The prediction of the equivalent permeability coefficient for the soil-cement diaphragm wall is important to optimize the engineering design.

**Figure 16:**
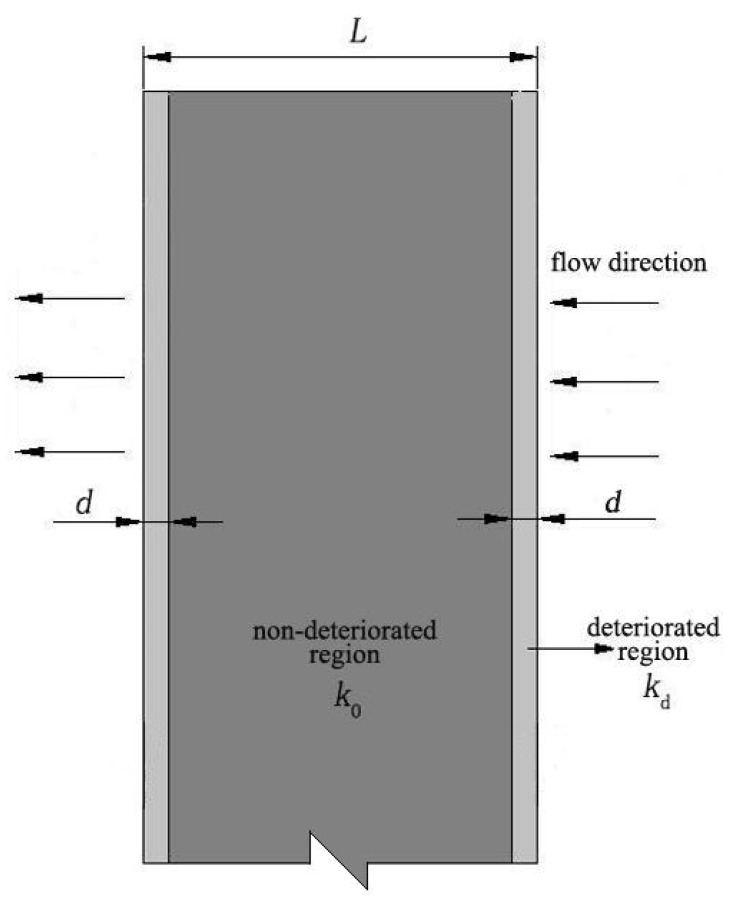
Deterioration of the diaphragm wall.

Figure 16 shows a deteriorated soil-cement diaphragm (*L* is the wall width and *d* is the deteriorated depth). According to the conservation of flow, Equation (15) can be obtained as follows:

(15) $$k_{d}i_{d}=k_{0}i_{0}=k_{c}i_{c},$$

where *k_d_* is the permeability coefficient of the deteriorated layer; *k_0_* is the permeability coefficient of non-deteriorated soil-cement; and *k_c_* is the equivalent permeability coefficient of the entire diaphragm wall.

The total head loss through the soil-cement diaphragm is the sum of the head losses through each part of the wall, so the following equation can be obtained:

(16) $$2i_{d}d+i_{0}(L-2d)=i_{c}L.$$

According to Equations (15) and (16), the equivalent permeability coefficient *k_c_* can be calculated as

(17) kc=k0kdL[2dk0+(L−2d)kd].

In Equation (17), *k_0_* is the permeability coefficient of the non-deteriorated soil-cement with a 90-day age; *d* and *k_d_* can be obtained according to Equations (13) and (14).

**Figure 17:**
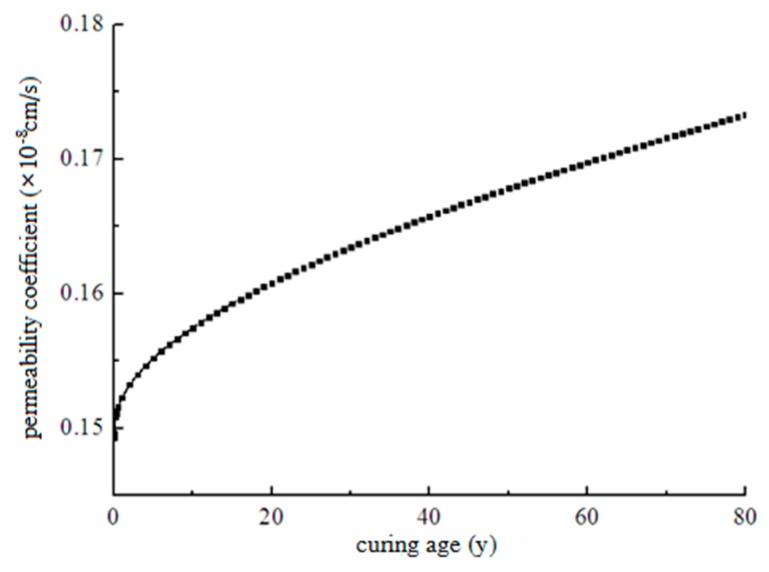
The time-history curve of the equivalent permeability coefficient of the diaphragm wall.

The time-history curve of the equivalent permeability coefficient of a 50 cm wide diaphragm wall can be obtained according to Equation (17), as shown in Figure 17. It is shown that the equivalent permeability coefficient initially increases linearly with time, but the growth rate slows with an increase in curing age.

## 7. Conclusions

Deterioration of soil-cement occurs in saline environments, leading to an increase in permeability. However, the equivalent permeability coefficient of a soil-cement specimen maintained in a corrosive laboratory curing environment cannot be directly applied to soil-cement anti-permeability designs because of the size effect. Therefore, this study modified the existing soil-cement permeability testing apparatus and proposed a new measurement method for the deteriorated layer permeability coefficient of soil-cement. This permeability coefficient can be obtained through a laboratory test and used in practical engineering. Using the marine dredger fill from Jiaozhou Bay in China as an example, a series of permeability tests of soil-cement deterioration layers with various cement contents were carried out, and the following conclusions were made:

(1) The growth rate of deterioration depths of soil-cement specimens gradually decreased with an increase in curing age. The deterioration depth increased with an increase in cement content.
(2) The growth rate of permeability coefficients increased with curing age and cement content. When the curing age was shorter, the permeability coefficient of soil-cement cured in seawater was smaller than that maintained in a curing room, but with increasing age, the permeability coefficient of soil-cement under immersion maintenance was higher than that maintained in a curing room.
(3) The evolution curve of the permeability coefficient of a deteriorated layer can be formulated as a logistic function.

## Figures and Tables

**Table 1:** Basic physical parameters of the marine dredger fill.

Specific Gravity of Soil Particle	Natural Moisture Content (%)	Plastic Limit (%)	Liquid Limit (%)
2.66	73.6	28.7	50.3

**Table 2:** Major ions content of the marine dredger fill.

**Ions**	${\mathrm{Mg}}^{2+}$	${\mathrm{Ca}}^{2+}$	${\mathrm{Cl}}^{-}$	${\mathrm{SO}}_{4}{}^{2-}$	Total Salinity
**Concentration(mg/L)**	303.9	714.0	12407.5	2401.5	26287.4

**Table 3:** Ions in the seawater.

**Ions**	${\mathrm{Cl}}^{-}$	${\mathrm{Na}}^{+}$	${\mathrm{Mg}}^{2+}$	${\mathrm{SO}}_{4}{}^{2-}$	$K^{+}$	${\mathrm{Ca}}^{2+}$	${\mathrm{Br}}^{-}$
**Content (g/L)**	16.7	9.4	1.2	2.1	0.34	0.35	0.05

**Table 4:** Fitting parameters.

Cement Content	*k_i_* (×10^−8^ cm/s)	*k_u_* (×10^−8^ cm/s)	*t_c_* (d)	p
7%	0.07	15.10	74.54	8.31
10%	0.03	10.81	75.72	7.82
15%	0.50	9.74	78.90	7.71
